# Biological and biomechanical evaluation of interface reaction at conical screw-type implants

**DOI:** 10.1186/1746-160X-2-5

**Published:** 2006-02-21

**Authors:** Andre Büchter, Ulrich Joos, Hans-Peter Wiesmann, László Seper, Ulrich Meyer

**Affiliations:** 1 Department of Cranio-Maxillofacial Surgery, University of Münster, Waldeyerstraße 30, D-48129 Münster, Germany; 2 Department for Cranio- and Maxillofacial Surgery, Heinrich-Heine-University, Moorenstr, 5, D-40225 Dusseldorf, Germany

## Abstract

**Background:**

Initial stability of the implant is, in effect, one of the fundamental criteria for obtaining long-term osseointegration. Achieving implant stability depends on the implant-bone relation, the surgical technique and on the microscopic and macroscopic morphology of the implant used. A newly designed parabolic screw-type dental implant system was tested in vivo for early stages of interface reaction at the implant surface.

**Methods:**

A total of 40 implants were placed into the cranial and caudal part of the tibia in eight male Göttinger minipigs. Resonance frequency measurements (RFM) were made on each implant at the time of fixture placement, 7 days and 28 days thereafter in all animals. Block biopsies were harvested 7 and 28 days (four animals each) following surgery. Biomechanical testing, removable torque tests (RTV), resonance frequency analysis; histological and histomorphometric analysis as well as ultrastructural investigations (scanning electron microscopy (SEM)) were performed.

**Results:**

Implant stability in respect to the measured RTV and RFM-levels were found to be high after 7 days of implants osseointegration and remained at this level during the experimented course. Additionally, RFM level demonstrated no alteration towards baseline levels during the osseointegration. No significant increase or decrease in the mean RFM (6029 Hz; 6256 Hz and 5885 Hz after 0-, 7- and 28 days) were observed. The removal torque values show after 7 and 28 days no significant difference. SEM analysis demonstrated a direct bone to implant contact over the whole implant surface. The bone-to-implant contact ratio increased from 35.8 ± 7.2% to 46.3 ± 17.7% over time (p = 0,146).

**Conclusion:**

The results of this study indicate primary stability of implants which osseointegrated with an intimate bone contact over the whole length of the implant.

## Introduction

The long-term success of osseointegrated implants in the treatment of completely and partially edentulous patients with a sufficient amount and quality of bone has been well documented in the literature [1-14]. Initial stability of the implant is, in effect, one of the fundamental criteria for obtaining long-term osseointegration. [4,6]. Achieving implant stability depends on the implant-bone relation, the surgical technique and on the microscopic and macroscopic morphology of the implant used.

The osseointegration mode of implants is influenced by the features of the implant system. Important aspects of a fast implant osseointegration include the need to achieve a primary congruence between the implant and the bone directly after insertion, the need to insert the implant with minimal surgical trauma and the capability of the implant surface to attach directly to the adjacent bone tissue. It has generally been thought in implant dentistry that osseointegration requires a healing period of at least 3 months in the mandible and 5 to 6 months in the maxilla [1-3,14,15]. The rationale for choosing a delayed loading period was that premature loading resulted in fibrous tissue encapsulation rather than direct bone apposition[4,6]. Nevertheless, several protocols for immediate and early loading have been presented and were found successful over the last two decades. According to Szmukler-Moncler et al. (2000) [16] two effective approaches can be used to reduce time between surgery and prosthetic reconstruction. One is to reduce micro-motion beneath the critical threshold by means of rigid fixation of loaded implants. The other possibility is to optimize the healing period before a safe functional loading can be exerted.

The importance of the implant geometries and surface characteristics, in an effort to achieve better bone anchorage, has been clear for a long time and, [4,17] in fact, various implant systems have been introduced over the past several years in order to achieve a faster bone integration [18]. In order to fasten the osseointegration process a new parabolic screw-type implant system was developed. The gross morphology of the implants was designed with the help of finite element analysis (FEA). The geometry of the implant was designed to allow micromovements of a magnitude between 500 and 3,000 μstrain in the loaded bone layer adjacent to the implant and to achieve a close congruency between the surgically created implantation bed and the implant surface direct after insertion [19-23].

We analysed in a combined approach the histological and biomechanical outcome of a new implant system. Biological investigations (histology, histomorphometry and scanning electron microscopy (SEM)) as well as biomechanical tests (resonance frequency measurements (RFM) and removal torque tests) were performed at early phases of implant/bone interaction in order to evaluate the time course of implant osseointegration.

## Materials and methods

### Implant System

**Figure 1:**
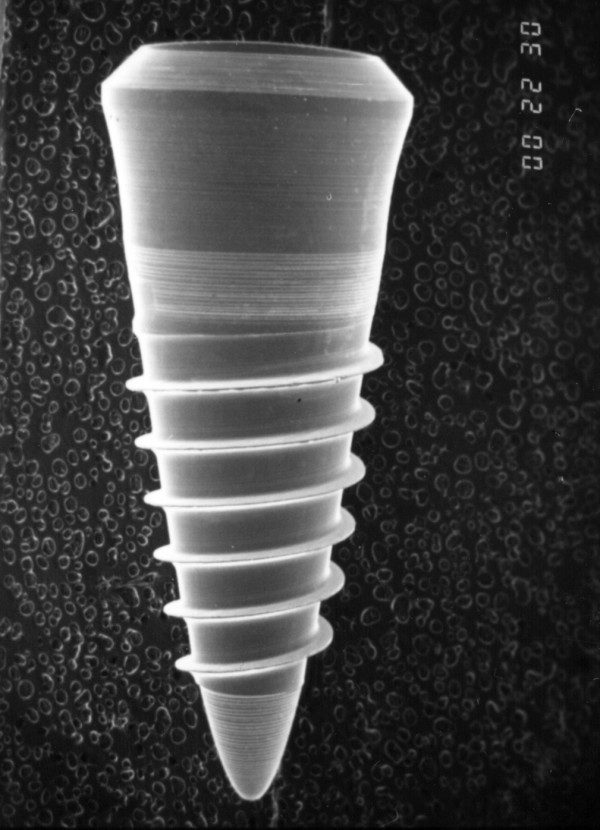
SEM of the implant used in this study (length 10 mm, shoulder diameter 4.1 mm). Microgrooves were located at the shoulder and tip of the implant.

**Figure 2:**
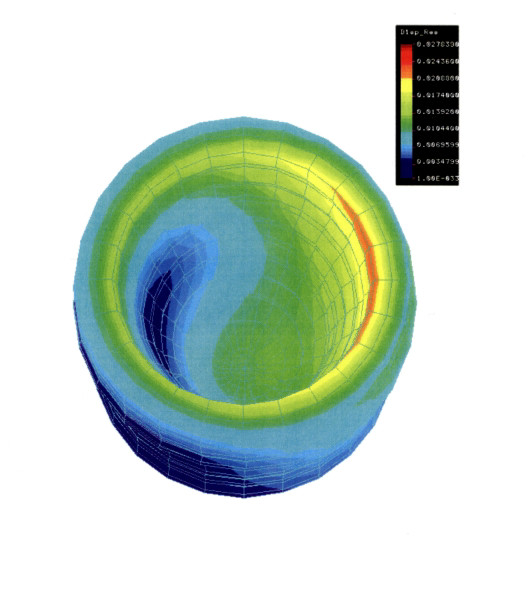
Finite element model of strain distribution under vertical load. The model corresponds to the implant and bone anatomy at the implant site.

The implants used in this study were newly developed parabolic screw-type implants (ILI) with a length of 10 mm and a diameter of 4.1 mm at the shoulder of the implant (Fig 1). The implants were made of pure titanium with a characteristic progressive thread design. The threads as well as the curvature of the implant provided a homogeneous strain distribution over the whole implant surface under vertical loading conditions (Fig 2), as revealed by finite element analysis [20]. The implants possess a microstructured texture of 20 – 30 μm deep grooves, where as the titanium surface it is smooth on a nanoscale level. The implant system consists of two parabolic burrs of different diameters and morphologies. The burrs are used subsequently to prepare the bony implantation bed. The diameter of the second burr is slightly smaller then the core diameter of the implant. Implants have a transversal core/thread relation of 1:1.2. Implant insertion is performed by manual tapping of the self cutting implants into the surgically created bony implantation bed.

### Experimental animals

**Figure 3:**
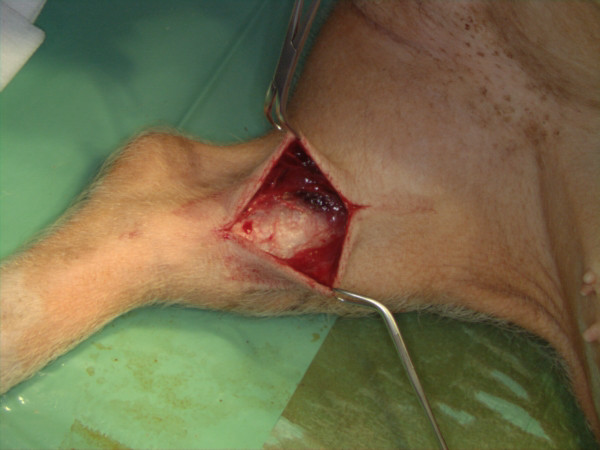
Scheme of implant placement (surgical procedure).

Eight male Göttinger minipigs, 14 to 16 months of age and with an average body weight of 35 kg were used in this study. A total of 40 implants were placed into the cranial and caudal part of the tibia condoyle (Fig 3). This study was approved by the Animal Ethics Committee of the University of Münster under the reference number G 38/2003.

### Surgical procedure

All surgery was performed under sterile conditions in a veterinary operating theatre. The animals were sedated with an intramuscular injection of ketamine (10 mg/kg), atropine (0.06 ml/kg) and stresnil (0.03 ml/kg). In the areas exposed to surgery 4 ml of local anaesthesia (2% lidocaine with 12.5 μg/ml epinephrine, Xylocain/ Adrenalin^®^, Astra, Wedel, Germany) was injected. The tibias were exposed by skin incisions and via fascial-periosteal flaps. Thereafter, the implants were placed in the cranial and caudal part of the tibia. The implant sites were sequentially enlarged with both drills according to the standard protocol of the manufacturer. Implants with 10 mm in length and 4,1 mm in diameter were inserted by using continuous external sterile saline irrigation to minimize bone damage caused by overheating. At the surgical site, the skin and the fascia-periosteum were closed in separate layers with single resorbable sutures (Vicryl^®^4-0, Ethicon, Norderstedt, Germany). Perioperatively, an antibiotic was administered subcutaneously (benzylpenicillin/dihydrostreptomycin, Tardomycel^®^, BayerVital, Leverkusen, Germany), 2.5 ml every 48 h for 7days. After placement, the shoulder of each implant was 1 mm below the ridge crest to allow circumferential bone growth. Resonace frequency measurements (RFM) (Osstell, Integration Diagnostics, Gothenburg, Sweden) were made for each implant at the time of fixture placement and after euthanasia [24-27]. The animals were inspected after the first few postoperative days for signs of wound dehiscence or infection and weekly thereafter to assess general health. Healing periods of 7 days and 28 days were allowed for half of the implants respectively. After 7 days and 28 days animals were sacrificed (4 minipigs each) with an overdose of T61 given intravenously. Following euthanasia, tibia block specimens containing the implants and surrounding tissues were dissected from all of the animals. The block samples were sectioned by a saw to remove unnecessary portions of bone and soft tissue and were prepared for the various investigations:

### Removal torque testing

The removal torque test was performed by applying a counter-clockwise rotation to the implant, around its axis at a rate of 0,1°/s according to the experimental set up of Li et al. 2002 [28]. For each implant the torque rotation curve was recorded. The removal torque was defined as the maximum torque (Nmm) on the curve. The interfacial stiffness was defined as the slope (Nm/degree of the torque-rotation curve) calculated from a linear regression analysis of the data between 0,5° and 3°.

### Resonance frequency measurements (RFM)

This method, as a non-destructive technique, evaluates the implant stability in term of interfacial stiffness. Resonance frequency measurements were made on each implant at the time of fixture placement and after the time of sacrifice (7 and 28 days) in all animals by attaching a 4-mm long standard transducer (Osstell, Integration Diagnostics, Gothenburg, Sweden) to the implant. The excitation sign was given over a range of frequencies (typically 5 kHz to 15 kHz with a peak amplitude of 1 V) and the first flexural resonance was measured [24-27]. The frequency responses of the system were measured for each implant.

### Scanning electron microscopy (SEM)

Block samples containing the implants were first divided into 2 halves, and then each sample was further dissected with a blade to obtain a sample containing the implant embedded in the alveolar bone and the corresponding bone sample detached from the implant (28 days after implant placement). Samples containing the implant were used for scanning electron microscopy (SEM). For SEM, glutaraldehyde-fixed specimens were critical point-dried. Samples were sputtercoated with gold for histological analysis. Specimens were examined under a fieldemission scanning electron microscopy (LEO 1530 VP, Oberkochen, Germany).

### Histomorphometry

The implants were removed together with the surrounding bone and fixed in Schaffer's solution (ethanol (96%), formaldehyde (37%), ratio: 2:1). The specimens were dehydrated in a graded series of ethanol. Thereafter, samples were embedded in methylmetacrylate (Technovit^®^7200, Heraeus Kulzer, Dormagen, Germany). Utilizing the 'sawing and grinding' technique, longitudinal sections were grounded to about 43–50μm for conventional microscopy (Exakt Apparatebau, Norderstedt, Germany). Five samples stained by Alizarin S 1% and Brilliant-Kresyl-blue 0,1% were prepared for each implant site. Histology was analysed by light microscopy (Zeiss, Axioplan 2, Göttingen, Germany).

Filters of wavelengths of 510–560 nm (green filter), 450–490 nm (blue filter), 355–425 nm (violet filter) and 340–380 nm (UV filter) (Zeiss, Göttingen, Germany) were utilized. The bone-to-implant contact ratio was defined as the length of bone surface border in direct contact with the implant (× 100 (%)). NIH-Imge software was used for image processing and analysis (National Institutes of Health, Bethesda, MD, USA)

### Statistical analysis

Mean values and standard deviations (SD) were calculated for RFM, removal torque testing, interfacial stiffness and bone-to-implant contact ratio. Multiple comparisons between all groups were performed using two-way analysis of variance and the t-test. Difference was considered significant when p < 0,05. All calculations were performed through the use of SPSS for Windows (SPSS Inc., Chicago, IL, USA).

## Results

### Clinical observation

All implants were anchored monocortically. At placement and during healing, the implants remained clinically immobile. The animals recovered well after surgery and no signs of infection were noted at any time during the observation period.

### Removal torque testing

Over the healing periods tested, the mean maximum torque of implants was 390.00 ± 148.32 Nmm after 7 days and 300.00 ± 69.22 Nmm after 28 days (Table 1). Statistically significant differences in the removal torque values were not observed between day 7 and day 28 (p = 0.351). The implant stiffness (Table 2), as assessed by the linear regression analysis, was higher after 7 days (0.3992 ± 0.063) than after 28 days (0.2648 ± 0.0257), but the difference was statistically not significant (p = 0.086).

**Table 1:** Removal torque values (Nmm) at two different healing periods

**Treatment group**	**7 days**	**28 days**	**Change within group**	**Change 7 days to 28 days**
**ILI**	390.00 ± 148.32	300.00 ± 69.22	90.00 ± 91.97 b	p = 0.351

**Table 2:** Implant-bone interfacial stiffness values (Nmm/degree) 7 and 28 days

**Treatment group**	**7 days**	**28 days**	**Change within group**	**Change 7 days to 28 days**
**ILI**	0.3992 ± 0.063	0.2648 ± 0.02257	0.1477 ± 0.039	p = 0.086

### Resonance frequency measurements

The RFM demonstrated no significant change in the resonance frequency responses during the 28 days the experimental period. Implants had a high primary stability as revealed by RFM (6029 ± 458 Hz and 6057 ± 423) directly after insertion. The implant stability remained at this baseline level through the experimental course (6257 ± 229 Hz at day 7 and 5885 ± 367 Hz at day 28) (Table 3 and 4).

**Table 3:** Resonance frequencies measurement (Hz) for 7 days

**Treatment group**	**0 day**	**7 days**	**Change within group**	**Change 0 day to 7 days to 7**
**ILI**	6029 ± 485	6257 ± 229	229 ± 203	p = 0.291

**Table 4:** Resonance frequencies measurement (kHz) for 28 days

**Treatment group**	**0 day**	**28 days**	**Change within group**	**Change 0 days to 28 days**
**ILI**	6057 ± 423	5885 ± 367	171 ± 211	p = 0.435

### SEM

**Figure 4:**
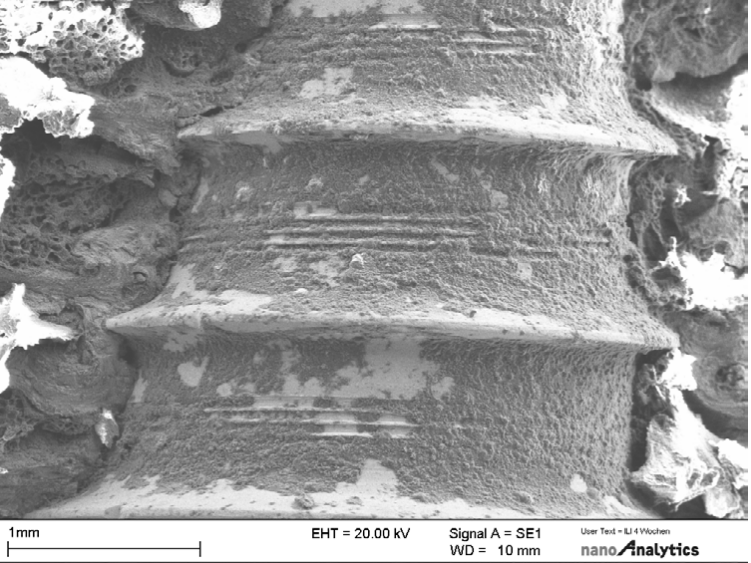
SEM of implantation sites in tibia specimens at different magnifications. Bone-implant interfaces are shown after 28 days of osseointegration.

**Figure 5:**
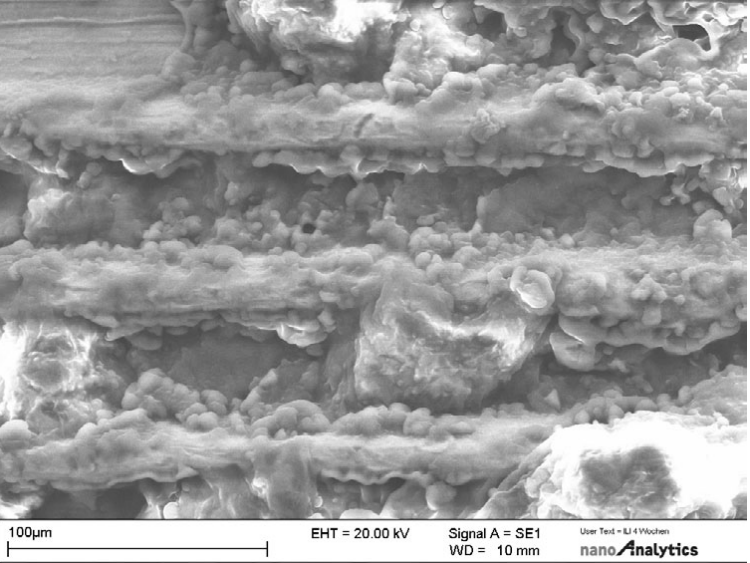
SEM of implantation sites in tibia specimens at different magnifications. Bone-implant interfaces are shown after 28 days of osseointegration.

Dissection of the implant-containing bone by a blade confirmed the clinical finding that the implants were well osseointegrated after 28 days. There was intimate bone contact over the whole length of the implant (Fig 4). Typically, endosteal bone covered the implant surface. Collagen fibres and osteoblasts made up the bulk of the adjacent tissue layer. The collagen fibres appeared to be predominantly oriented perpendicular to the implant surface in the bulk bony tissue (Fig 5). Cells, extracellular matrix proteins, and mineralized bone tissue were in direct contact with the implant. In contrast to the collagen fibres in the original bone, which were oriented perpendicular to the implant, newly synthesized collagen in the vicinity of the surface appeared to form a felt-like matrix parallel to the surface. Intimate bone contact was presented at the neck of implants. Typically, cells (osteoblasts) were firmly attached to the implant surface. Probe processing by sample fracturing for the electron microscopic investigations suggested that the bond between the implant and the adjacent bone layer seemed to mimic the bond in the bone tissue itself. On the implant surface, cells and extracellular matrix remained attached following separation from the enveloping bone.

### Histological and histomorphometric measurements

**Figure 6:**
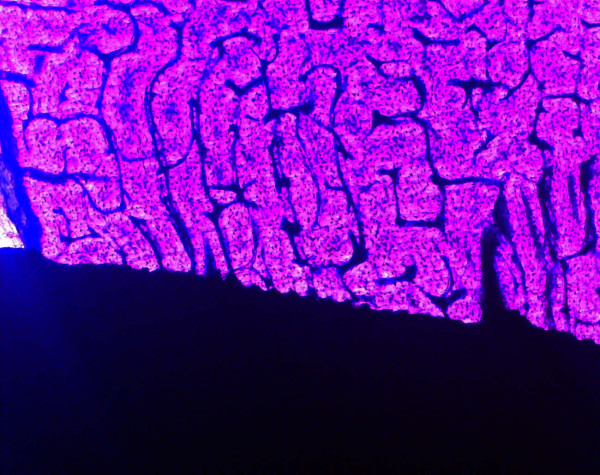
Light micrographs showing a direct cortical bone/implant interface (after a healing period of 28 days; magnification × 40).

**Figure 7:**
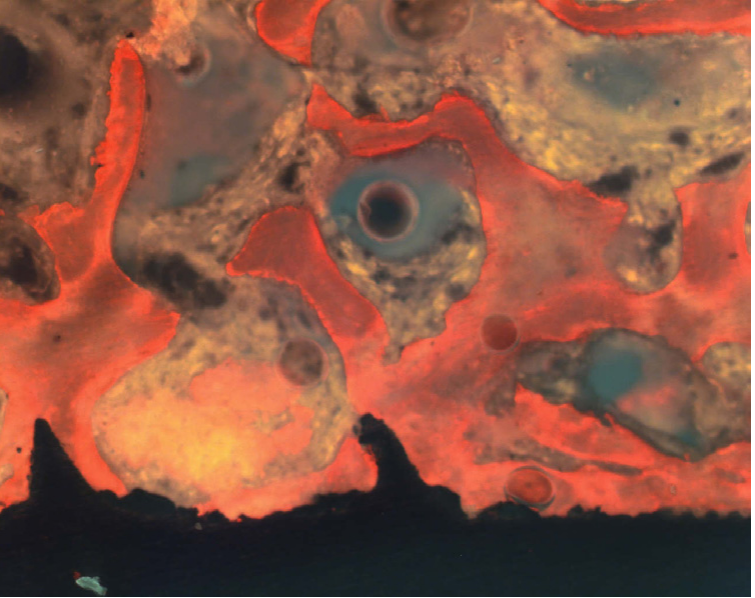
Laminar bone structure as revealed by fluorescence microscopy without labelling (healing period 28 days; magnification × 40).

**Figure 8:**
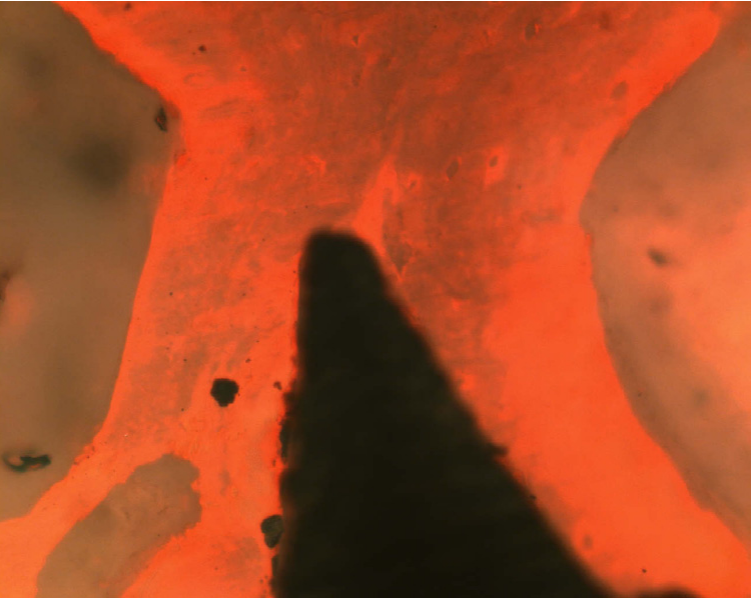
Laminar bone structure as revealed by fluorescence microscopy without labelling (healing period 28 days; magnification × 60).

Direct bone-to-implant contact could be achieved during the healing period. There were no signs of inflammation. Histological analysis of the bone/implant interface revealed an intimate contact between the titanium surface and the bony implantation bed. At the bone-titanium interface, a thin tissue layer stained with Alizarin S and Brilliant-Kresyl-blue was seen in some areas coming into direct contact with the titanium. The bony apposition revealed a laminar structure containing individual osteocytes and Haversian canals at the neck of implants (Fig 6). The laminar bone demonstrated also an intimate contact between spongiosal trabecula and the implant surface at the body of implants (Fig 7 and 8). Quantitative histomorphometric analysis revealed an enhanced bone-to-implant contact for every healing period. After 7 days the bone-to-implant contact ratio was 35.8 ± 7.2% and after 28 days the bone-to-implant contact ratio was 46.3 ± 17.7%, but the difference in the bone to implant contact did not reach a level of significance (table 5). A direct bone – implant contact was documented especially at the cortical bone area (neck of implants) after 7 and 28 days (Fig 6).

**Table 5:** Bone-to-implant contact ratio (%)

**Treatment group**	**7 days**	**28 days**	**Change within group**	**Change 7 days to 28 days**
**ILI**	35.82 ± 7.2	46.33 ± 17.69	10.51 ± 6.79	p = 0.146

## Discussion

Insights into cellular processes occurring at the implant/bone interface have contributed much to an understanding of osseointegration. The understanding of the complex bone/implant interactions at different levels will provide an opportunity to evaluate and produce implants with specific and desired biologic responses [29-31]. The implant system used in this study was designed to allow implants to have a high primary stability and a direct bone/implant contact over the whole implant surface directly after insertion. Various studies emphasise that the mode of implant osseointegration and stability is dependent to a large extent on the gross and ultrastructural implant design [4-7]. However, the role of implant geometry and surface structuring in affecting early tissue healing and implant stability cannot be determined only from histological or biomechanical observations. The dynamics of bone physiology can also not be evaluated several weeks post-implantation after long term bone remodelling has occurred. Therefore, early bone responses have to be considered when the influence of implant geometry and surface structuring on interface formation is under investigation.

Evaluation of implant stability can be performed by destructive (removal torque tests) or alternative by non-destructive measures (RFA). Alternatively, the architecture of the tissue/implant interface is visualised by histological techniques (light microscopy) or ultrastructural analysis (electron microscopy). The different biomechanical and biological approaches, except for the RFA determinations, exclude each other because of the sample preparation. Therefore, a combined probe sampling and preparation was done in this study to give a better insight into the morphological and functional features of interfacial tissue formation.

The histological overview of the bone/implant features in the present study demonstrates a congruency between the implant and the surrounding bone tissue. A direct contact between titanium and bone was visible over the whole surface area of the implant directly after insertion and during the experimental period. Bone was in close contact even towards the rim and the groove areas of the microstructered titanium surface. One underlying reason for the direct bone/implant contact found in this study may be the three dimensional geometric relation between the final burr and the implant in combination with the self cutting properties of the implant. Bone tissue has under ideal circumstances isotropic elastic properties. Despite the fact that mechanically influenced bone probably exhibits a more complex situation, it behaves elastic up to a distinct rate of deformation. A number of in vivo studies confirmed that minor bone deformations will not disturb but in contrast will strengthen bone tissue [31]. The finding of a high primary congruency between parabolic shaped implants and the peri-implant tissue may be explained by an insertion into a slightly widened bony implantation bed.

Various modifications of the implant surface, as a second approach to improve the bone to implant ratio, have also been utilised. To increase the overall implant surface various surface modifications were introduced in implant design and fabrication. The importance of implant surface properties for the subsequent osseointegration was first pointed out by Albrektsson etal. (1981) [4]. At a state-of-the-art meeting on tissue integration held in 1985, the importance of implant surface properties for biological responses were further emphasised in a consensus agreement that stated; 'surface properties are important for and may be used to facilitate tissue integration [32]. However, a number of questions have followed regarding the mode of the surface properties of titanium implants, especially during early stages of implant osseointegration. The increase in the overall implant surface was demonstrated by various authors to be accompanied by an enhanced bone to implant ratio at later stages of bone/ implant interaction [17,33]. The range of early bone-implant-contact in this study (36 – 46 %) corresponds well to the data (40% at the surface of TIO2-blasted and machine-prepared implants) reported by Ericsson et al. (1984) [33] after two month of osseointegration, indicating a good primary bone/implant contact.

Excellent adaptation of the host bone to titanium surfaces was observed also on an ultrastructural level in a comparable manner as reported after insertion of self cutting screws in calvaria bone by Sowden and Schmitz (2002) [34]. It was demonstrated that when self -tapping screws were placed in loading or non-loading positions the long-term histology showed that the amount of bone tissue around implants was maintained in both situations [35]. In agreement with the histological findings of the present study, Murai et al. (1996) [36] demonstrated also a thin 20–50 μm sized layer in some places at the implant surface, preferentially at the spongiosal part of the implant interface. The electron microscopical observations at day 28 of implant/bone interaction demonstrate that not only mineralized bone tissue contacts the surface but that viable osteoblasts are also attached firmly to the titanium surface. Our results are in agreement with findings of Lavos-Valereto et al. (2001) [37] who demonstrated an intimate contact between mineralised matrix and cells and the titanium implants on an SEM level in the early and late post-implantation time.

The RFM and removal torque values were found to be comparable towards stability determination of implants after various times of osseointegration [38-42]. The removal torque tests and the RFM of this study confirm the high primary stability of implants directly after placement (RFM) and after 7 days of implant osseointegration (RFM and removal torque values). Implant stability after 4 weeks of osseointegration reached values of the baseline level, indicating high primary implant stability. The results of the presented biomechanical evaluation methods are in agreement with previous trials, demonstrating a direct relationship between removal torque determinations and resonance frequency measurement [9-12]. The range of RFM found in this study corresponds well to the data reported by Meredith et al. (1997) [26]. Whereas the relation between removal torque measurements and RFM is not fully understood at present, the outcome of both techniques probably relates to the complex biomechanical properties of the bone adjacent to the implant to a high degree. In the presented study we found a correlation between the histomorphometric and biomechanical measurements, indicating that the combined histological and biomechanical approach reflect the biological situation of peri-implant bone. As primary stability is necessary to establish mechanical rest, which is one of the essential factors for the development of osseointegration [9,42], and additionally the gross implant geometry leads to a homogenuous strain distribution in loaded peri-implant bone [22], the implant system incorporates the prerequisites for applying immediate loading protocols. It was demonstrated in additional animal experimental studies that immediate loading of such implants can be performed without disturbance of the early osseointegration process [21-23].

In this study the bone implant contact ratio increases by 10% over a month period, but the RTV and RFM of the implants stay almost stable. This implies that the biomechanical properties of the healing interface (interface stiffness) does not increase at the clinical level and it is probably not the macrodesign but the microtopography of implants that leads to this result. Taking the test period into account, the cortical bone surrounding the implant neck conceals the improvement in RFM analyses, and since the biomechanical properties of the healing bone tissue is very low, in comparison with cortical bone, the RTV does not increase.

## Conclusion

The present study indicates a high primary stability of biomimetrically designed implants, based on an intimate bone contact over the whole length of the implant.

## Competing interests

The author(s) declare that they have no competing interests.

## Authors' contributions

AB designed the study, searched the database, extracted the data. UJ helped with the study design and analysis. HPW had analysis the histological probes and UJ developed the implant design.
